# Doctor for a day: the impact of a health professions enrichment program on self-efficacy

**DOI:** 10.3389/fmed.2025.1511405

**Published:** 2025-02-25

**Authors:** Celeste Coler, Kareena Patel, A. J. Balatico, Kumhee Ro, Joshua Villarreal, Nora Coronado, Estell Williams

**Affiliations:** ^1^School of Medicine, University of Washington, Seattle, WA, United States; ^2^College of Education, University of Washington, Seattle, WA, United States; ^3^College of Nursing, Seattle University, Seattle, WA, United States; ^4^School of Pharmacy, University of Washington, Seattle, WA, United States; ^5^Department of Surgery, University of Washington, Seattle, WA, United States

**Keywords:** diversity, self-efficacy, mentorship, healthcare, medicine, underrepresented, outreach

## Abstract

**Introduction:**

Health profession enrichment programs for underrepresented minority students are crucial to supporting students’ interest in healthcare careers and improving preparedness for important academic and professional milestones. Doctor for a Day, an enrichment program at the University of Washington School of Medicine, hosts monthly events where underrepresented kindergarten-12th grade students are exposed to careers in medicine by healthcare professionals from diverse backgrounds.

**Methods:**

This study investigates to what extent participation in Doctor for a Day programming improves self-efficacy using a survey study of 958 students who attended at least one Doctor for a Day event between 2017 and 2023.

**Results:**

Using an evaluation tool composed of six questions, our results demonstrate that participation in Doctor for a Day programming increases self-efficacy and interest in medicine as a career. Analysis of these results found significant differences in responses based on grade level, with students in high school demonstrating the largest improvement in self-efficacy.

**Discussion:**

These findings underscore the value of such enrichment programs and offer insights for the development of similar initiatives.

## 1 Introduction

A diverse healthcare workforce is critical to improving the quality of care offered to diverse populations (1, 2). This is supported by a growing body of evidence that highlights the connection between increased diversity among healthcare professionals and the enhanced delivery of culturally appropriate care to diverse patient populations (1, 3–8). While the racial and socioeconomic diversity in many healthcare professions has improved in recent years, students who are the first in their family to attend college and those from racially and ethnically minoritized groups, rural regions, and low socioeconomic backgrounds remain grossly underrepresented across the healthcare workforce (9, 10). For example, in the last decade, Black and Latinx individuals have comprised only 5 and 5.8% of the physician workforce despite representing 12.8 and 18.4% of the general United States population, respectively, (10). In 2022, the National Nursing Workforce Survey reported that 6.2% of registered nurses identify as Black or African American and 6.9% identify as Latinx (11). The United States Bureau of Labor Statistics reported similar disparities in 2022 among dentists, physical therapists, and occupational therapists (12). In terms of socioeconomic representation, 25% of medical students report parental income in the top 5% of United States households while students of families in the lowest quintile make up only 6% of medical students (13).

The explanation for these disparities is multifactorial and includes challenges due to systemic and institutional racism, inadequate or biased career advising, high costs of education, and difficulty accessing required courses (14–17). The disproportionately low number of physicians from backgrounds underrepresented in medicine also contributes to fewer opportunities for race-concordant mentorship among underrepresented youth, trainees, and faculty (18, 19). This is particularly concerning, as research has consistently shown that race-concordant mentorship is important for learning and professional development, and can even inform the medical specialty students ultimately pursue (20–22). Furthermore, race-concordant mentorship has been shown to minimize perceived challenges and address feelings of isolation (23). Enrichment programs for students traditionally underrepresented in medicine help overcome these barriers to medicine by providing science education, clinical exposure, and race-concordant mentorship. They also represent an evidence-based approach to promoting health equity and matriculating diverse student populations into healthcare professional training programs (24).

There is tremendous variation between pre-health enrichment programs nationwide. Parsons et al.’s review of physician pipeline and pathway programs highlights the diversity of current enrichment initiatives, which target various age groups, help with the development of different skill sets, and vary in time commitment. Some programs are short-term workshops (e.g., mock interviews, application workshops, financial literacy seminars, sample lectures), while others require longitudinal engagement (e.g., standardized test preparation, residential summer programs). These programs also employ a range of instructional methods, including problem-based learning, role-playing, and hands-on didactics (25).

There are various ways to evaluate enrichment programming, each offering unique insights into program effectiveness and impact. One common method of evaluation is assessing changes in self-efficacy, which is defined as one’s belief in their ability to achieve a goal and overcome challenges. In recent years, the construct of self-efficacy has become increasingly used to evaluate health profession enrichment programs (22, 26), as it has been shown that increased self-efficacy is associated with greater resilience in the face of academic challenges, increased likelihood to take on challenging tasks, and increased preparedness for a healthcare career (26–28). Social theories such as Bandura’s social cognitive theory also propose that student beliefs about personal capabilities may play a role in future choices such as career decisions (29, 30). Additional evaluation methods involve assessing changes in academic performance, skill development, or participant engagement. Research indicates that many enrichment programs linked to improvements in grade point average, retention rates, academic and research knowledge, as well as personal and professional growth (25, 31–36).

University of Washington’s Doctor for A Day (DFAD) is an enrichment program for kindergarten-12^th^ grade (k-12) students traditionally underrepresented in health careers that aims to address disparities within the healthcare workforce and introduce youth to healthcare careers. The objective of this study is to determine whether participation in Doctor for a Day increases participants’ self-efficacy toward a career in medicine.

## 2 Methods

### 2.1 Program description

DFAD was designed to address barriers that underrepresented students face when exploring careers in healthcare. DFAD provides mentorship, opportunities to develop professional and interpersonal skills, knowledge on applying to graduate biomedical programs, and exposure to scientific topics relevant to medical careers. In DFAD’s monthly event, students from backgrounds traditionally underrepresented in health careers spend an afternoon engaging in hands-on activities such as suturing, physical exam skills, and ultrasound, led by medical students, residents, and faculty from various health specialties. Students also learn about disparities in healthcare and innovative solutions to the current challenges in medicine. Each event focuses on a specific organ system or disease process, providing variety for students who choose to attend multiple events.

The DFAD model is novel in its approach, in that it utilizes service learning to provide mentorship at multiple levels. Our curriculum allows students to interact and learn from individuals from diverse backgrounds who are at various points in their training and medical careers. Additionally, medical students learn from faculty, fellows, and residents as they work together to develop informative and interactive workshops. Attendings, residents, and fellows also benefit by giving back to local communities, further strengthening their community connections. Not only is learning facilitated at all levels, but inter-professionalism is emphasized through the representation of diverse healthcare careers and specialties. This creates a cycle of giving, learning, trust, and community building that has a far-reaching impact.

### 2.2 Participants

All Washington State k-12 students are eligible to participate in DFAD programming. K-12 students include students between the ages of 5–18. All participants participate in the same workshops regardless of age, and students are not separated based on age or grade level. Many of the participants are affiliated with one of DFAD’s community partners, including Gear Up, College Success Foundation, Spin Girls, Rainier Scholars, Africatown Center for Excellence and Innovation, Boys and Girls Club, Young Men’s Christian Association, and multiple schools and school districts across Washington State. Community partnerships are critical to DFAD because they help facilitate information sharing about specific community needs to best support individual and group learning and help with the recruitment and transportation of students.

### 2.3 Volunteers and mentorship

Many healthcare careers are represented among the volunteers including medical students, residents, attendings, and allied health professionals (i.e., physical and occupational therapists, respiratory therapists, etc.). Volunteers are responsible for leading small cohorts of students through hands-on activities and answering students’ questions during panel discussions at each event. These interactive activities are meant to provide insight into a healthcare field, medical specialty, or disease process and introduce students to the interdisciplinary nature of healthcare.

### 2.4 Evaluation

Immediately following participation in a DFAD event, participants complete an anonymous post-participation survey developed by researchers from Seattle University and the University of Washington that assesses changes in self-efficacy and attitudes towards healthcare careers, participant demographics, and program and curricular feedback (Supplementary material S1).

The instrument used here was developed to measure the construct of self-efficacy within the domains of “understanding” and “ability.” Understanding was measured using the statements “I better understand what it takes to go to a medical or other health care professional school,” “I better understand what a doctor does,” and “I better understand what other health care professionals (e.g., nurses, dentists, PT and OT, etc.) do.” Ability was measured using the statements “I am more likely to go to college,” “I am more likely to pursue a career in the health care professions (e.g., doctor, nurse, dentist, etc.),” “I feel that having a career as a healthcare professional 116 is an achievable goal for me”. Each question response used a 5-point Likert scale.

In the survey, students also reported their race, gender identity, and grade level. Students between grades k-5 (elementary school) were between the ages of 5–9, students between grades 6–8 (middle school) were between the ages of 10–14, and students between grades 9–12 (high school) were between the ages of 15–18.

This study was deemed exempt by the University of Washington’s Institutional Review Board because participants incurred minimal risk. Informed consent from parents and legal guardians and assent from all students was obtained during registration for the workshop and in the post-participation survey.

### 2.5 Analysis

Statistical analysis of demographic data including frequencies and means were performed on survey responses from 2017 to 2023 using SPSS v.24. Statistical analysis of self-efficacy Likert-score was conducted using two-sample t-tests and ANOVA tests. Effect sizes were expressed as Hedges’s g. Significance was denoted by *p* < 0.05.

## 3 Results

Between 2017 and 2023, 958 students attended at least one of 29 DFAD events and completed a post-participation survey (Table 1). Of the 958 students, 26.8% were Asian, 34% were Black or African American, 16.4% were Latinx/Hispanic, 3.4% were Middle Eastern, 4.4% were mixed-race, 1% were American Indian or Alaska Native, 5.7% were white, and 8.8% did not disclose. Among all participants, 0.3% were in college, 73.4% were in high school, 22.8% were in middle school, 4.2% were in elementary school, and 0.4% did not disclose. Additionally, 76.6% identified as female and 21.2% identified as male. Additional participant demographics were recorded, including the level of parent education and the use of free or reduced lunch programs (Supplementary material S2).

**Table 1:** Workshops hosted from the 2016–2017 school year to 2022–2023 school year not including virtual events hosted during the COVID-19 pandemic.

Year	Number of events	Number of participants
2016–2017	4	77
2017–2018	6	218
2018–2019	5	188
2019–2020	7	226
2022–2023	4	249

**Figure 1:**
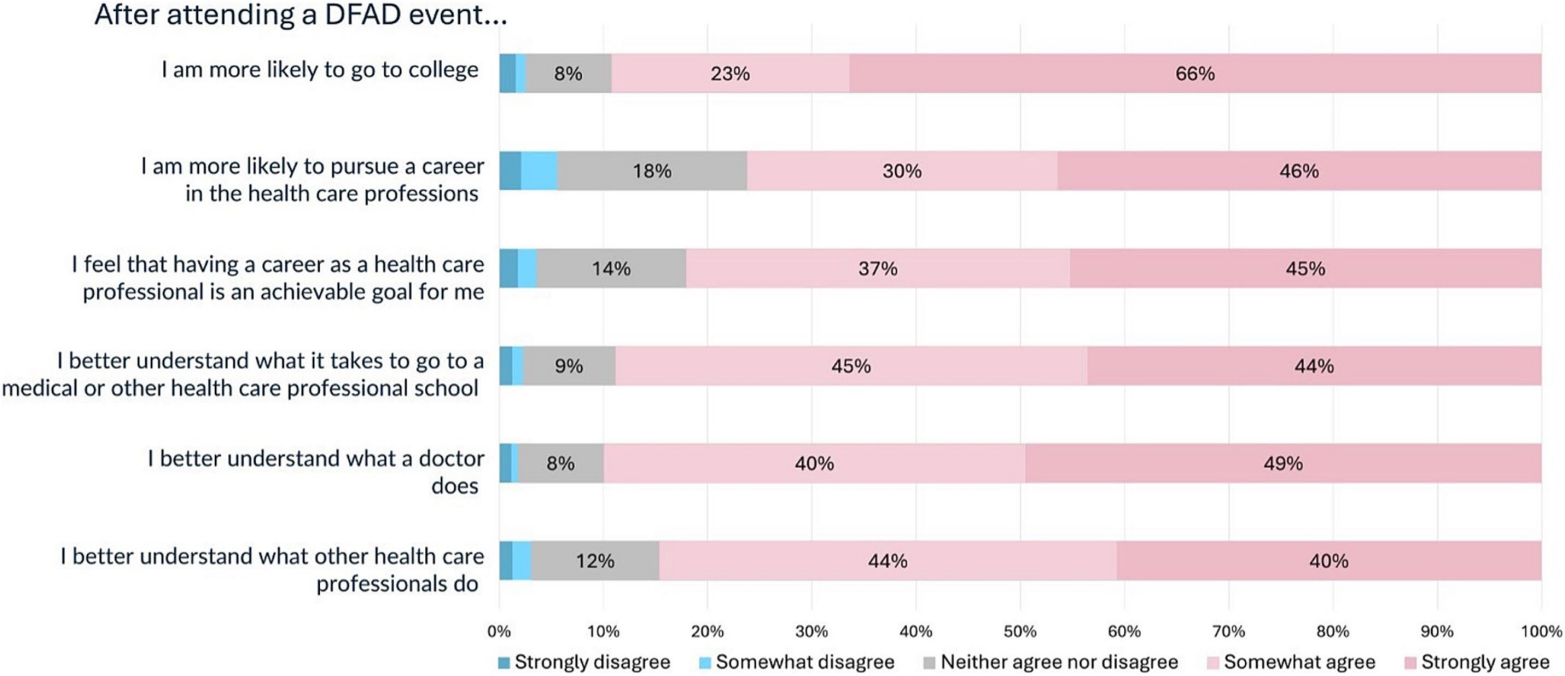
Likert-scale responses to questions evaluating self-efficacy after attending a DFAD event.

Likert scales were used to record the responses to six questions assessing students’ perceived ability, understanding, and interest in healthcare professions following participation in DFAD programming (Figure 1). 89% agreed or strongly agreed with the statement “I am more likely to go to college,” 76% agreed or strongly agreed with the statement “I am more likely to pursue a career in the health care professions (e.g., doctor, nurse, dentist, etc.),” 82% agreed or strongly agreed with the statement “I feel that having a career as a health care professional is an achievable goal for me,” 89% agreed or strongly agreed with the statement “I better understand what it takes to go to a medical or other health care professional school,” 89% agreed or strongly agreed with the statement “I better understand what a doctor does,” and 84% agreed or strongly agreed with the statement “I better understand what other health care professionals (e.g., nurses, dentists, physical therapists and occupational therapists, etc.) do.”

ANOVA tests showed that there were significant differences in responses by age for the following questions: “I am more likely to pursue a career in the health professions” with a small effect size between the elementary and high school groups (Hedge’s g = 0.44), “I better understand what it takes to go to a medical or other health care professional school” with a small effect size between the elementary vs. high school groups (Hedge’s g = 0.40), and “I better understand what other healthcare professionals (e.g., nurses, dentists, physical therapists, occupational therapists) do” (*p* < 0.001) with a small effect size between the middle vs. high school groups (Hedge’s g = 0.30) (Table 2).

**Table 2:** Self-efficacy questionnaire by grade level (elementary school: ages 5–9, middle school: ages 10–14, high school: ages 15–18).

Question	Elementary School Mean (Variance)	Middle School Mean (Variance)	High School Mean (Variance)
I am more likely to go to college	4.36 (0.67)	4.50 (0.69)	4.52 (0.68)
I am more likely to pursue a career in the health care professions (e.g., doctor, nurse, dentist, etc.)	3.80 (1.11)	4.00 (1.09)	4.23 (0.89)
I feel that having a career as a health care professional is an achievable goal for me	4.15 (0.45)	4.09 (0.87)	4.23 (0.65)
I better understand what it takes to go to a medical or other health care professional school	4.02 (1.19)	4.20 (0.59)	4.33 (0.55)
I better understand what a doctor does	4.33 (0.69)	4.34 (0.61)	4.33 (0.55)
I better understand what other health care professionals (e.g., nurses, dentists, physical therapists and occupational therapists, etc.) do	4.15 (0.80)	4.14 (0.69)	4.37 (0.58)

Race was not associated with a significant difference in the response to “I am more likely to go to college” (*F* = 1.51, *p* = 0.17), “I am more likely to pursue a career in the health care professions (e.g., doctor, nurse, dentist, etc.)” (*F* = 1.17, *p* = 0.32), “I feel that having a career as a health care professional is an achievable goal for me” (*F* = 0.96, *p* = 0.45), “I better understand what it takes to go to a medical or other health care professional school” (*F* = 0.81, *p* = 0.56), “I better understand what a doctor does” (*F* = 1.02, *p* = 0.41), and “I better understand what other health care professionals (e.g., nurses, dentists, physical therapists, and occupational therapists, etc.) do” (*F* = 1.94, *p* = 0.072). Therefore, race did not significantly affect changes in self-efficacy (Table 3).

**Table 3:** Self-efficacy questionnaire by self-reported race.

Question	American Indian, Native American, or Alaska Native Mean (Variance)	Asian Mean (Variance)	Black/ African American Mean (Variance)	Latinx/Hispanic Mean (Variance)	Middle Eastern Mean (Variance)	White Mean (Variance)	Mixed Mean (Variance)	*F*-statistic
I am more likely to go to college	4.40 (1.16)	4.59 (0.59)	4.54 (0.71)	4.46 (0.67)	4.46 (0.90)	4.28 (0.86)	4.64 (0.43)	1.51
I am more likely to pursue a career in the health care professions (e.g., doctor, nurse, dentist, etc.)	4.0 (1.56)	4.24 (0.72)	4.18 (1.08)	4.05 (1.03)	4.22 (1.07)	4.17 (0.95)	3.90 (0.72)	1.18
I feel that having a career as a health care professional is an achievable goal for me	4.2 (1.73)	4.18 (0.74)	4.30 (0.83)	4.16 (0.77)	4.31 (0.93)	4.25 (0.65)	4.07 (0.60)	0.95
I better understand what it takes to go to a medical or other health care professional school	4.40 (0.71)	4.29 (0.54)	4.32 (0.60)	4.21 (0.65)	4.44 (0.71)	4.41 (0.43)	4.36 (0.38)	0.81
I better understand what a doctor does	4.4 (0.49)	4.37 (0.51)	4.36 (0.63)	4.39 (0.65)	4.56 (0.64)	4.46 (0.48)	4.52 (0.40)	1.02
I better understand what other health care professionals (e.g., nurses, dentists, physical therapists and occupational therapists, etc.) do	4.30 (0.68)	4.23 (0.54)	4.20 (0.77)	4.08 (0.64)	4.45 (0.79)	4.35 (0.61)	4.43 (0.45)	1.94

## 4 Discussion

Survey responses demonstrated that DFAD programming is utilized by underrepresented minority students with multiple social and economic barriers to healthcare careers. Over half of the DFAD participants received free and reduced lunch, a proxy for low socioeconomic status (37, 38). Moreover, a third of students had parents with a high school diploma as their most advanced degree, and half of the students did not know someone with a job in healthcare. These findings indicate that many students attending DFAD events may lack the necessary support networks and guidance to navigate graduate education and careers in healthcare.

Additionally, responses to the six self-efficacy questions suggest that participation in DFAD programming increases students’ self-efficacy, interest in medicine as a career, and understanding of different healthcare careers. These findings are consistent with similar enrichment programs including the Access Summer Scholars Program at the University of Pennsylvania which showed an improved understanding of the physician identity and increased preparedness for medical school following program participation (39). The Vanderbilt Summer Science Academy similarly found that program participation was associated with an improved understanding of post-graduate programs and a greater sense of belonging in the science and medical community (40).

To date, few studies assess how student age or grade may influence program effectiveness. In this study, grade level appeared to play an important role in influencing perceived self-efficacy after DFAD attendance despite all students receiving the same curriculum regardless of age. Our findings suggest that DFAD participation resulted in the greatest increase in self-efficacy for high school students compared to middle and elementary school students. Although self-efficacy was greatest among high school students, research has also shown that without early intervention with enrichment programming in elementary school, underrepresented students may lose interest and confidence in science and math by their teenage years, underscoring the importance of enrichment programs that include elementary-aged students (41). When designing curriculums for DFAD events, volunteers must balance the needs of younger students, who benefit most from programs that foster interest and self-confidence in the sciences, with those of older students, who benefit from programs that prepare them academically for challenging science courses (41).

Very few studies have assessed whether the effect of an enrichment program differs by student race. An evaluation of the Center for Disease Control and Prevention Public Health Scholars Program found a difference in program influence by racial and ethnic background, however, this study does not include whether these differences were statistically significant (42). By demonstrating that there were no statistically significant differences in survey responses by self-reported race, this study addresses a critical knowledge gap.

### 4.1 Future directions

Our assessment of DFAD programming would benefit from pre-participation surveys assessing baseline interest in healthcare careers and self-efficacy, as well as long-term follow-up. Although the anonymity of DFAD’s surveys makes long-term follow-up challenging, long-term follow-up is a valuable way of evaluating DFADs’ influence on high school, undergraduate, and graduate school matriculation and ultimate career choice.

In the future, conducting interviews and focus groups with current and past participants may allow for a more nuanced exploration of the factors influencing participants’ experiences, motivations, challenges, and perceptions. Qualitative research may also benefit our assessment of DFAD by contextualizing and validating quantitative findings. Additionally, future surveys should collect information on the number of DFAD events students have participated in to determine whether consistent participation has a greater impact on the behaviors and beliefs being studied. We have adapted our surveys for the 2024–2025 academic year to collect this information. In addition, the adapted study is being reviewed using exploratory factor analysis to test for the reliability of the adapted survey questions.

Finally, studies have shown that volunteering can decrease burnout and stress and improve the quality of life among healthcare professionals and trainees (43–45). Still, data on the effect of enrichment programs on volunteers’ burnout is limited. Since DFAD programming supports mentorship between volunteers at various points in their healthcare professional training, assessing the impact of DFAD on volunteers’ job satisfaction, burnout, and quality of life may address a large gap in the literature.

### 4.2 Limitations

One limitation of this study is the use of an evaluation tool that has not yet been validated, which may affect the reliability of the findings. Without formal validation, it is unclear whether the evaluation tool consistently measures the intended constructs. Future program evaluation should prioritize the use of a validated tool to enhance the generalizability of the results.

This cohort study is also limited due to the lack of information about the participants’ baseline characteristics prior to participating in DFAD programming. The distribution of pre-participation surveys that assess baseline interest in healthcare professions and self-efficacy would provide useful information regarding the impact of DFAD on self-efficacy and attitudes toward healthcare careers.

### 4.3 Conclusion

This study adds to the growing literature on the impact of enrichment programming on self-efficacy among underrepresented students interested in medicine. The findings of this study demonstrate that participation in DFAD increases participants’ self-efficacy toward a career in medicine, with high school students showing the greatest improvement in their self-efficacy. These findings highlight the program’s potential to support the career aspirations of underrepresented students interested in medicine and improve access to medicine as a career.

## Data Availability

The original contributions presented in the study are included in the article/Supplementary material, further inquiries can be directed to the corresponding author.
