# Supplementary material for: Doctor for a day: the impact of a health professions enrichment program on self-efficacy
Source: Front Med (Lausanne). 2025 Feb 25;12:1511405. doi: 10.3389/fmed.2025.1511405 (PMC11893586; doi:10.3389/fmed.2025.1511405)
Supplement: Supplementary file 1 [file Data_Sheet_1.pdf]

Doctor for a Day Survey

* Required

Please complete this survey after participating in today's Doctor for a Day event.

1. Please select each race/ethnicity that represents how you identify:
*

- American Indian, Native American, or Alaska Native
- Asian
- Black or African American
- Latinx/Hispanic
- Middle Eastern
- Mixed race
- White
- Other

2. Please select the gender identity that best represents how you identify: *

- Woman
- Man
- Non-binary/third gender

3. Are you affiliated with any of the following programs? *

- ◯ Gear Up
- ◯ College Success Foundation
- ◯ Spin Girls
- ◯ Ranier Scholars
- ◯ Africatown
- ◯ None/Other

4. Highest level of education of your parents or guardians *

- ◯ Some or all of elementary school
- ◯ Some or all of middle school
- ◯ Some high school
- ◯ High school diploma
- ◯ Trade school
- ◯ Some college, no degree
- ◯ Associate's degree
- ◯ Bachelor's degree
- ◯ Graduate degree

5. Do you have any family members or close friends in a health profession? *

- ◯ Yes
- ◯ No

6. If yes, what do they do?

7. Do you get free or reduced lunch? *

- ◯ Yes
- ◯ No

8. After attending this even please select the response that best describes your reaction to the following statements: *

	Strongly agree	Somewhat agree	Neither agree nor disagree	Somewhat disagree	Strongly disagree
I am more likely to go to college	◯	◯	◯	◯	◯
I am more likely to pursue a career in the health care professions (e.g. doctor, nurse, dentist, etc.)	◯	◯	◯	◯	◯
I feel that having a career as a health care professional is an achievable goal for me	◯	◯	◯	◯	◯
I better understand what it takes to go to a medical or other health care professional school	◯	◯	◯	◯	◯
I better understand what a doctor does	◯	◯	◯	◯	◯
I better understand what other health care professionals (e.g. nurses, dentists, physical therapists and occupational therapists, etc.) do	◯	◯	◯	◯	◯
