# Supplementary material for: Doctor for a day: the impact of a health professions enrichment program on self-efficacy
Source: Front Med (Lausanne). 2025 Feb 25;12:1511405. doi: 10.3389/fmed.2025.1511405 (PMC11893586; doi:10.3389/fmed.2025.1511405)
Supplement: Supplementary file 2 [file Table_1.docx]

Supplementary Materials S2. Participant demographics

| Demographic | 2016-2017 | 2017-2018 | 2018-2019 | 2019-2020 | 2022-2023 | Total % of Participants |
| --- | --- | --- | --- | --- | --- | --- |
| Race and ethnicity | | | | | | |
| American Indian or Alaska Native | 2 (2.6%) | 2 (0.9%) | 0 | 1 (.4%) | 5 (2%) | 10 (1%) |
| Asian | 17 (22%) | 61 (28%) | 50 (26.6%) | 49 (21.7%) | 79 (32.7%) | 256 (26.8%) |
| Black or African American | 34(44.2%) | 79 (36.2%) | 79 (42.%) | 59 (26.1%) | 73 (29.3%) | 324 (34%) |
| Latinx/Hispanic | 14(18.2%) | 36 (16.5%) | 24 (12.8%) | 37 (16.3) | 45 (18.1%) | 156 (16.4%) |
| Middle Eastern | 1(1.3%) | 7 (3.2%) | 2 (1.1%) | 18 (8%) | 4 (1.6%) | 32 (3.4%) |
| Mixed | 3 (3.9%) | 13 (6%) | 11(5.9%) | 12 (5.3%) | 3 (1.2%) | 42 (4.4%) |
| White | 1 (1.3%) | 8 (3.7%) | 5 (2.7%) | 15 (6.6%) | 25 (10%) | 54 (5.7%) |
| Did not disclose | 5 (6.5%) | 12 (5.5%) | 17 (9%) | 35 (15.5%) | 15 (6%) | 84 (8.8%) |
| Gender | | | | | | |
| Male | 13 (16.9%) | 36 (16.5%) | 45 (23.9%) | 65 (28.8%) | 44 (17.7) | 203 (21.2%) |
| Female | 63 (81.8%) | 179 (82.1%) | 141 (75%) | 153 (67.7%) | 198 (79.5%) | 734 (76.6%) |
| Other | 1(1.3%) | 3 (1.4%) | 2 (1.1%) | 8 (5.5%) | 6 (2.4%) | 20 (2.1%) |
| Age | | | | | | |
| Elementary School (K-5) | 0 | 8 (3.7%) | 4 (2.1%) | 27 (12%) | 1 (.4%) | 40 (4.2%) |
| Middle School (6-8) | 26 (33.8%) | 48 (22%) | 28 (14.9%) | 97 (42.9%) | 19 (7.6%) | 218 (22.8%) |
| High school (9-12) | 51 (66.2%) | 162 (7.41%) | 155 (82.5%) | 110 (48.7%) | 225 (90.4%) | 703 (73.4%) |
| Undergraduate | 0 | 0 | 1 (.5%) | 0 | 2 (.8%) | 3 (.3%) |
| Did not disclose | 0 | 0 | 0 | 2 (.9%) | 2 (.8%) | 4 (.4%) |
| Parent Education | | | | | | |
| Some of high school | 19 (24.7%) | 37(17%) | 30(16%) | 25 (11.1%) | 38 (15.3%) | 149 (15.6%) |
| High school diploma | 14 (18.2%) | 22(10.1%) | 22 (11.7%) | 21 (9.3%) | 23 (9.2%) | 102 (10.7%) |
| Trade school | 2 (2.6%) | 6(2.75%) | 1 (.5%) | 4 (1.8%) | 1 (.4%) | 14 (1.5%) |
| Some college, no degree | 5 (6.5%) | 31 (14.2%) | 24 (12.7%) | 25 (11.1%) | 27. (10.8%) | 112 (11.7%) |
| Associate degree | 4 (5.2%) | 13 (6%) | 17 (9%) | 15 (6.6%) | 8 (3.2%) | 57 (6%) |
| Bachelor's degree | 6 (7.8%) | 41 (18.8%) | 34 (18.1%) | 52 (23%) | 52 (20.9%) | 185 (19.3%) |
| Graduate degree | 19 (24.7%) | 49 (22.5%) | 43 (22.9%) | 54(23.9%) | 74 (29.7%) | 239 (24.95%) |
| Did not disclose | 8 (10.4%) | 16 (7.3%) | 14 (7.5%) | 20 (8.9%) | 21 (8.4%) | 79 (8.25%) |
| Receives free or reduced lunch | | | | | | |
| Yes | 42 (54.6%) | 113 (51.8%) | 116 (61.7%) | 114 (50.4%) | 150(60.2%) | 535 (55.9%) |
| No | 27 (35.06% | 75 (34.4%) | 48 (25.5%) | 98 (43.4%) | 91 (36.6%) | 339 (35.4%) |
| Did not disclose | 8(10.4%) | 29 (13.3%) | 23 (12.2%) | 13 (5.8%) | 7 (2.8%) | 80 (8.4%) |
| Has a family member of close friend in a health profession | | | | | | |
| Yes | 41 (53.3%) | 116 (53.2%) | 90 (47.9%) | 126 (55.8%) | 127 (51%) | 500 (52.2%) |
| No | 36 (46.8%) | 99 (45.4%) | 91 (48.4%) | 93 (41.2%) | 116 (46.6%) | 435 (45.4%) |
| Did not disclose | 0 | 3(1.4%) | 7 (3.7%) | 6 (2.7%) | 6 (2.4%) | 22 (2.3%) |
